# Uropathogenic *Escherichia coli* virulence characteristics and antimicrobial resistance amongst pediatric urinary tract infections

**DOI:** 10.25122/jml-2021-0148

**Published:** 2022-05

**Authors:** Narjes Alfuraiji, Amal Al-Hamami, Maysaa Ibrahim, Hassan Khuder Rajab, Balsam Waleed Hussain

**Affiliations:** 1. Department of Pharmacology, College of Medicine, University of Kerbala, Kerbala, Iraq; 2. Department of Pediatrics, College of Medicine, Aliraqia University, Baghdad, Iraq; 3. Department of Pharmacology, College of Medicine, Tikrit University, Tikrit, Iraq; 4. Department of General Surgery, Al-Yarmouk Teaching Hospital, Baghdad, Iraq

**Keywords:** Uropathogenic *Escherichia coli*, virulence factors, antimicrobial resistance, pediatrics, UTIs – Urinary Tract Infections, *E. coli* – *Escherichia coli*, UPEC – Uropathogenic *Escherichia coli*, PCR – Polymerase Chain Reaction, *sfa* – S fimbriae, *afa* – A fimbrial adhesin, *cnf1* – cytotoxic necrotizing factor 1, *pap* – pyelonephritis-associated pil.

## Abstract

Uropathogenic *Escherichia coli* (UPEC) harbors virulence factors responsible for bacterial adhesion and invasion. In addition, the bacterium is accountable for the occurrence of pediatric urinary tract infections globally and is becoming problematic due to the emergence of antimicrobial resistance. The current research investigated UPEC prevalence, virulence characteristics, and antimicrobial resistance in pediatric urinary tract infection (UTI). 200 urine specimens were taken from hospitalized pediatric patients who suffered from UTIs. *E. coli* was recovered from urine specimens using the microbial culture. Disc diffusion method was used to assess antimicrobial resistance and polymerase chain reaction (PCR) to assess the virulence factors distribution amongst the UPEC bacteria. Seventy-five out of 250 (30.00%) urine samples were positive for the UPEC bacteria. The UPEC prevalence amongst pediatric patients was 25.83% and 33.84%, respectively. UPEC bacteria harbored the maximum resistance toward gentamicin (45.33%), ampicillin (44.00%), and ciprofloxacin (40.00%). Cytotoxic necrotizing factor 1 (*Cnf1)* (53.33%) and pyelonephritis-associated pil (*pap)* (42.66%) were the most frequently identified virulence factors amongst the UPEC bacteria. The high prevalence of UPEC isolates harboring antimicrobial resistance and virulence factors suggest that diseases caused by them need more expansive healthcare monitoring with essential demand for novel antimicrobials.

## INTRODUCTION

Urinary tract infections (UTIs) are still considered a significant infectious disease globally. UTIs are referred to infection and inflammation of different sites of the urinary system, particularly the bladder, ureter, urethra, and kidneys [1]. UTIs are concerning health issues affecting 150 million individuals globally yearly [2]. It is also a common and complicated infection among children, particularly infants. Global estimates revealed that both girls (8%) and boys (2%) experienced no less than one UTI episode, with 12–30% recurrent cases [3].

Uropathogenic *Escherichia coli* (UPEC) bacteria are the most emerging cause of UTIs globally [4]. The bacterium is armed with diverse kinds of putative virulence factors, including S fimbriae (*sfa*), A fimbrial adhesin (*afa*), cytotoxic necrotizing factor 1 (*cnf1*), and pyelonephritis-associated pili (*pap*) [5]. Most act as adhesive and invasive factors that trigger precise signaling pathways causing renal inflammation and damage. Their activities in the cases of pyelonephritis, urethritis, and cystitis have been reported [6, 7].

The emergence of antimicrobial resistance increases the importance of UPEC as most isolates harbor significant resistance toward diverse classes of antimicrobial agents, especially tetracyclines, penicillins, cephalosporins, aminoglycosides, fluoroquinolones, and macrolides [8]. As a result, most therapeutic options fail, leading to increased hospital stays and treatment costs.

Given the high importance of UPEC UTIs among pediatric patients and their uncertain epidemiological aspects, this research was performed to evaluate the prevalence, antimicrobial resistance, and virulence characteristics of UPEC bacteria recovered from pediatric patients suffering from UTIs.

## MATERIAL AND METHODS

### Urine specimens

From February to December 2020, 200 urine specimens were taken from pediatric patients (male and female, <3 years old) hospitalized at the Department of Urosurgery, Al-Yarmouk Teaching Hospitals, Baghdad, Iraq. All samples were collected from volunteers and written informed consents were signed by their parents. Pediatrics were hospitalized due to UTIs. Midstream urine was taken through sterile conditions to reduce possible microbial and artifactual contaminations. Urine specimens were taken using sterile glass tubes (10 mL) and immediately transported to the laboratory at 4°C [9].

### E. coli identification

According to Bailey and Scott's technique [9], *E. coli* bacteria were isolated from the urine specimens. Briefly, urine specimens were cultured on nutrient agar (NA), MacConkey agar (MCA), sheep blood agar (BA), and eosin-methylene blue (EMB) agar (Merck, Germany) and incubated at 37°C for 24h. Lactose-positive (pink color) colonies in the MCA and green colonies with metallic polish in the EMB agar media were nominated as suspected colonies. *E. coli* isolates were identified by Gram-staining and numerous biochemical tests [9].

### Antimicrobial resistance pattern

Instructions announced by the Clinical and Laboratory Standard Institute (CLSI) were applied [10]. Mueller–Hinton agar (Merck, Germany) was used for *E. coli* culture. Diverse antimicrobial disks, such as cefotaxime (30 µg/disk), ciprofloxacin (5 µg/disk), gentamicin (10 µg/disk), ofloxacin (5 µg/disk), meropenem (10 µg/disk), ampicillin (10 µg/disk), trimethoprim-sulfamethoxazole (25 µg/disk), norfloxacin (10 µg/disk), amikacin (30 µg/disk), and nalidixic acid (30 µg/disk) were placed on media. Microbial media with placed disks were incubated (24 h at 37°C). For this purpose, bacterial concentrations were adjusted to 0.5 McFarland standard [11–13]. *E. coli* ATCC 25922 was applied as a control.

### DNA extraction and Polymerase Chain Reaction (PCR)

Tryptic Soy Broth (Merck, Germany) was used for *E. coli* growth before DNA extraction. DNA extraction kit (Thermo Fisher Scientific, Germany) was applied. The NanoDrop (NanoDrop, Thermo Scientific, USA) device was applied to assess extracted DNA quantitatively. The qualitative assessment of extracted DNA was performed using agarose gel electrophoresis (2%).

The most important virulence factors associated with the UPEC bacteria were detected using the PCR. Table 1 reveals the PCR circumstances [14, 15]. Eppendorf Mastercycler (Hamburg, Germany) device was applied for the amplification. PCR products were then electrophoresed (1.5% agarose gel contained ethidium bromide and 120 V/208 mA). Positive (positive DNA samples of each gene) and negative (PCR-grade water (Thermo Fisher Scientific, Germany)) controls were applied to monitor the findings of the PCR.

**Table 1:** PCR procedures used to detect virulence factors [14, 15].

Genes	Primers (5'-3')	PCR product (bp)	Thermal cycles	Volume (50μL)
***Afa***	F: GCT GGG CAG CAA ACT GAT AAC TCT C	750	**1 cycle** 3 min: 95°C	PCR buffer 10X: 5 μL Mgcl2: 2 mM dNTP: 200 μM Primer F: 0.5 μM Primer R: 0.5 μM Taq DNA polymerase: 1.5 U DNA: 5 μL
	R: CAT CAA GCT GTT TGT TCG TCC GCC G			
***Sfa***	F: CTC CGG AGA ACT GGG TGC ATC TTA C	410	**30 cycles** 60 s: 94°C 30 s: 63°C 90 s: 72°C	
	R: CGG AGG AGT AAT TAC AAA CCT GGC A			
***Pap***	F: GCA ACA GCA ACG CTG GTT GCA TCA T	336		
	R: AGA GAG AGC CAC TCT TAT ACG GAC A			
***Cnf1***	F: AAG ATG GAG TTT CCT ATG CAG GAG	498	**1 cycle** 8 min: 72°C	
	R: TGG AGT TTC CTA TGC AGG AG			

### Data analysis

Data collected were analyzed using SPSS/22.0. Qualitative data were examined using the chi-square test and Fisher's exact 2-tailed test. A P-value less than 0.05 was determined as a significance level.

## RESULTS

### UPEC prevalence

Table 2 reveals the UPEC prevalence amongst urine specimens collected from male and female pediatric patients. Seventy-five out of 200 (37.50%) urine samples were positive for the UPEC bacteria. The UPEC prevalence amongst the urine specimens collected from male and female pediatric patients was 34.44% and 40.00%, respectively. Amongst all examined age groups, 6 months to 1-year-old male (48.00%) and female (53.33%) pediatric patients harbored the highest prevalence of UPEC bacteria. There was a significant difference in UPEC prevalence between male and female pediatric patients (P<0.05). Furthermore, a significant difference was obtained in the UPEC prevalence between different age groups in pediatrics (P<0.05).

**Table 2:** UPEC distribution amongst examined urine specimens.

Urine specimens (Age groups)		N. collected specimens	N. specimens positive for UPEC (%)
**Male**	**6 months–1 year**	25 12 (48.00)	
	**1–2 years**	35	10 (28.57)
	**2–3 years**	30	9 (30.00)
	**Total**	90	31 (34.44)
**Female**	**6 months–1 year**	30	16 (53.33)
	**1–2 years**	35	14 (40.00)
	**2–3 years**	45	14 (31.11)
	**Total**	110	44 (40.00)
**Total**		200	75 (37.50)

### Antimicrobial resistance

Table 3 discloses the UPEC antimicrobial resistance toward diverse antimicrobial agents. UPEC bacteria harbored the maximum resistance toward gentamicin (45.33%), ampicillin (44.00%), and ciprofloxacin (40.00%). However, the lowest resistance rates were obtained toward meropenem (4.00%), norfloxacin (22.66%), and nalidixic acid (22.66%). UPEC isolates of female and 2-3-year-old pediatric patients harbored a higher resistance toward antimicrobial agents (P<0.05).

**Table 3:** UPEC resistance pattern toward antimicrobial agents.

Specimens (N. UPEC isolates)		N. isolates harbored resistance toward each antimicrobial (%)									
		Cef	Cip	Gen	Ofl	Mrp	Amp	Trsul	Nor	Amk	Nal
**Male**	**6 months–1 year (12)**	3 (25.00)	4 (33.33)	5 (41.66)	3 (25.00)	-	4 (33.33)	3 (25.00)	3 (25.00)	3 (25.00)	2 (16.66)
	**1–2 years (10)**	3 (30.00)	3 (30)	4 (40)	2 (20)	-	4 (40)	2 (20)	2 (20)	3 (30)	3 (30)
	**2–3 years (9)**	5 (55.55)	4 (44.44)	5 (55.55)	2 (22/22)	1 (11/11)	5 (55.55)	3 (33/33)	2 (22/22)	4 (44.44)	2 (22/22)
	**Total (31)**	11 (35.48)	11 (35.48)	14 (45.16)	7 (22.58)	1 (3.22)	13 (41.93)	8 (25.80)	7 (22.58)	11 (35.48)	7 (22.58)
**Female**	**0 months–1 year (16)**	4 (25.00)	5 (31.25)	6 (37.50)	4 (25.00)	-	5 (31.25)	4 (25.00)	3 (18.75)	5 (31.25)	3 (18.75)
	**1–2 years (14)**	6 (42.85)	7 (50.00)	6 (42.85)	4 (28.57)	1 (7.14)	7 (50.00)	6 (42.85)	4 (28.57)	6 (42.85)	3 (21.42)
	**2–3 years (14)**	8 (57.14)	7 (50.00)	8 (57.14)	3 (21.42)	1 (7.14)	8 (57.14)	5 (35.71)	3 (21.42)	6 (42.85)	4 (28.57)
	**Total (44)**	18 (40.90)	19 (43.18)	20 (45.45)	11 (25.00)	2 (4.54)	20 (45.45)	15 (34.09)	10 (22.72)	17 (38.63)	10 (22.72)
**Total (75)**		29 (38.66)	30 (40.00)	34 (45.33)	18 (24.00)	3 (4.00)	33 (44.00)	23 (30.66)	17 (22.66)	28 (37.33)	17 (22.66)

Cef – cefotaxime (30 μg/disk); Cip – ciprofloxacin (5 μg/disk); Gen – gentamicin (10 μg/disk); Ofl – ofloxacin (5 μg/disk); Mrp – meropenem (10 μg/disk); Amp – ampicillin (10 μg/disk); Trsu – trimethoprim-sulfamethoxazole (25 μg/disk); Nor – norfloxacin (10 μg/disk); Amk – amikacin (30 μg/disk); Nal – nalidixic acid (30 μg/disk).

### Virulence factors distribution

Table 4 discloses the UPEC virulence factor profile. *Cnf1* (53.33%) followed by *pap* (42.66%) were the most frequently identified virulence factors amid the UPEC bacteria. In contrast, the lowest distribution was obtained for *sfa* (16.00%) and *afa* (13.33%), respectively. UPEC isolates of female pediatric patients harbored higher virulence factors distribution (P<0.05).

**Table 4:** UPEC virulence factor profile.

Specimens (N. UPEC isolates)		N. isolates harbored resistance toward each virulence factor (%)			
		*afa*	*sfa*	*pap*	*Cnf1*
**Male**	**6 months–1 year (12)**	-	1 (8.33)	2 (16.66)	4 (33.33)
	**1–2 years (10)**	2 (20)	1 (10)	6 (60)	5 (50)
	**2–3 years (9)**	2 (22.22)	2 (22.22)	4 (44.44)	6 (66.66)
	**Total (31)**	4 (12.90)	4 (12.90)	12 (38.70)	15 (48.38)
**Female**	**6 months–1 year (16)**	1 (6.25)	2 (12.50)	4 (25.00)	4 (25.00)
	**1–2 years (14)**	2 (14.28)	3 (21.42)	6 (42.85)	8 (57.14)
	**2–3 years (14)**	3 (21.42)	3 (21.42)	12 (85.71)	13 (92.85)
	**Total (44)**	6 (13.63)	8 (18.18)	20 (45.45)	25 (56.81)
**Total (75)**		10 (13.33)	12 (16.00)	32 (42.66)	40 (53.33)

## DISCUSSION

About 8% of 1-month to 11 years old children have experienced a UTI. Additionally, about 30% of pediatric patients experience repeated UTIs throughout the first year of life [16]. Furthermore, it was estimated that about 650 million dollars are paid every year in the United States to treat UTIs [17]. UPEC bacteria are accountable for 90% of pyelonephritis cases, particularly in children [16]. As a result, UPEC bacteria have a high clinical standing in pediatric UTIs.

The present survey revealed that the prevalence of UPEC bacteria in the urine samples of pediatric patients was 37.50%, which was lower than those reported in Nepal (68.40%) [18], Saudi Arabia (75.70%) [19], and Qatar (32.40%) [20], and higher than those of Uganda (10.00%) [21], and Ethiopia (25.34%) [22]. The prevalence of UPEC was higher in females than males. This finding might be due to the wide and short urethra in the female, which increases the penetration and spread of bacteria [23, 24]. Additionally, 0-1-year-old pediatric patients had the highest prevalence of UPEC strains, which may be due to infections in the hospital environment at birth and their subsequent visits.

Our findings showed a relatively low UPEC resistance rate toward commonly used antimicrobials. The reason for this finding is probably the lack of antimicrobials prescription for pediatric patients. In this regard, a high UPEC antimicrobial resistance was reported in samples collected from adult patients [8, 25]. Mishra et al. (2016) [26] stated that the UPEC bacteria isolated from paediatric UTIs in India harboured the maximum resistance rate toward amikacin (31.00%), gentamicin (36.00%), netilmicin (31.00%), amoxiclav (36.00%), ampicillin (39.00%), piperacillin (36.00%), piperacillin/tazobactam (38.00%), ceftriaxone (36.00%), ceftazidime (30.00%), cefuroxime (31.00%), levofloxacin (38.00%), norfloxacin (36.00%), ofloxacin (36.00%), co-trimoxazole (31.00%), and nitrofurantoin (28.00%) antimicrobials. Ramos et al. (2011) [27] mentioned that amoxicillin/clavulanic acid and ampicillin resistance was predominant among Iranian, Australian, and Swedish uropathogens. From a global meta-analysis view [28], the maximum UPEC resistance rate obtained toward ampicillin was 53.40%, followed by co-trimoxazole (30.20%) and trimethoprim (23.60%), respectively. Furthermore, this rate reached 8.20% for co-amoxiclav, 2.40% for ceftazidime, 2.10% for ciprofloxacin, and 1.30% for nitrofurantoin, which was lower than our reports.

Uropathogenic *E. coli* causes the majority of UTIs in both inpatients and outpatients pediatrics. The severity of infection relies on the presence and activity of some putative virulence factors. Infection initiation depends on the UPEC adhesion into the renal epithelial cells. Fimbrial factors (*afa, sfa*, and *pap*) are among the essential agents for UPEC adhesion to the renal epithelium and subsequent damages [29, 30]. Toxins are other essential virulence factors arbitrating UPEC's host cell invasion, dissemination, and persistence [31]. The *cnf1* virulence factor is supposed to act by the iron release of red blood cells, phagocytic cell dysfunction, and renal tissue cytotoxicity [5]. UPEC dissemination and persistence depend on the *cnf1* activation [5]. Of 75 UPEC bacteria isolates in the current survey, 13.34%, 16.00%, 42.66%, and 53.33% exhibited *afa, sfa, pap*, and *cnf1* virulence factors, respectively, which may show their high pathogenicity. Boost prevalence of *afa, sfa, pap* and *cnf1* virulence factors amongst the UPEC bacteria was also reported in India [32] and Mexico [33]. The present study was limited to the lack of urine sample assessment of healthy pediatric patients as a control group, the low number of collected samples, and the lack of molecular assessment of antibiotic resistance genes.

## CONCLUSION

In conclusion, virulent and resistant UPEC strains are considered a predominant cause of UTIs among Iraqi pediatrics. The simultaneous presence of antimicrobial resistance and virulence factors might upsurge the pathogenicity of strains. According to findings, gentamicin, ampicillin, and ciprofloxacin prescription cannot effectively be controlled and treat the UPEC's UTIs in Iraq. However, further surveys should assess other epidemiological features of the UPEC in UTIs.
